# Thyroid Cancer: Epidemiology, Classification, Risk Factors, Diagnostic and Prognostic Markers, and Current Treatment Strategies

**DOI:** 10.3390/ijms26115173

**Published:** 2025-05-28

**Authors:** Alicja Forma, Karolina Kłodnicka, Weronika Pająk, Jolanta Flieger, Barbara Teresińska, Jacek Januszewski, Jacek Baj

**Affiliations:** 1 Department of Forensic Medicine, Medical University of Lublin, ul. Jaczewskiego 8b, 20-090 Lublin, Poland; 62108@student.umlub.pl (W.P.); 56555@student.umlub.pl (B.T.); 58441@student.umlub.pl (J.J.); 2 Department of Correct, Clinical, and Imaging Anatomy, Medical University of Lublin, Jaczewskiego 4, 20-090 Lublin, Poland; 62450@umlub.edu.pl (K.K.); jacek.baj@umlub.pl (J.B.); 3 Department of Analytical Chemistry, Medical University of Lublin, Chodźki 4A, 20-093 Lublin, Poland; jolanta.flieger@umlub.pl

**Keywords:** papillary thyroid cancer, follicular thyroid cancer, medullary thyroid cancer, anaplastic thyroid cancer, epidemiology, thyroid cancer, thyroid carcinoma

## Abstract

Thyroid cancer (TC) invariably remains the most prevalent endocrine cancer in the world. Major histological forms of TC include papillary (PTC), follicular (FTC), medullary (MTC), and anaplastic thyroid carcinoma (ATC), each of which has a unique clinical and molecular profile. The incidence rate of TC is higher in females, and unfortunately, it has tended to increase over the last several years. Yet the treatment of advanced or aggressive TC forms has improved recently because of developments in immunotherapy and targeted medicines, including PD-1 inhibitors and tyrosine kinase inhibitors (e.g., lenvatinib, sorafenib). Imaging, fine-needle aspiration biopsies, and molecular testing are implemented in the diagnostic process, e.g., in search of mutations that might affect prognosis and provide the most successful treatment option. Chemotherapy, immunotherapy, radioactive iodine therapy (RAI), surgery (such as a total thyroidectomy), and molecularly targeted therapies are currently standard treatment modalities in TC. Optimizing patient outcomes requires better diagnostic precision and individualized treatment regimens based on the genetic profile and tumor subtype. To improve survival and quality of life, it is critical to comprehend the complex etiology of TC and the changing therapeutic landscape.

## 1. Introduction

Thyroid cancer (TC) remains the most common endocrinological cancer worldwide, with a disturbingly increasing incidence rate over recent years [1,2]. The reason for this is not only a greater awareness of this malignancy and in-depth diagnosis leading to the early detection of less typical clinical symptoms but also its increasing occurrence due to a continually changing world and environmental threats associated with this. Environmental factors that are currently distinguished as potential risk factors for TC include the increasing usage of environmental chemicals as well as exposure to heavy metals, radiation, and air pollution [3,4,5,6,7]. Being exposed to the abovementioned factors constitutes an increasing global problem that might result in an increasing incidence of various endocrinological diseases and malignancies, including TC. An increase in the TC occurrence rate is associated with countries with a high or very high Human Development Index (HDI), where TC accounts for 91% of new cases [8]. In the following narrative review, we aimed to summarize the most recent knowledge regarding TC, including information about its epidemiology and changes in its trends, causes, prognostic and diagnostic factors, classification, and—most importantly—the current updates on TC treatment strategies and novel therapeutic approaches.

## 2. Epidemiology of Thyroid Cancer

Currently, TC is ranked as the 7th most prevalent cancer worldwide, with a mortality rate ranked as 24th highest among all cancers [9]. TC is three times more common in females than in males; the less aggressive histologic subtypes are more prevalent in females, while the more aggressive ones are equally frequent in both genders [10]. TC is often diagnosed at a younger age compared to any other adult cancer; the mean age at which TC is diagnosed is 51 [11]. Clinicopathological manifestations of TC differ depending on the age of the patient; e.g., younger patients more commonly present with lymph node metastasis, neurovascular, or capsular invasion, indicating that younger individuals usually present a more aggressive course of this malignancy [12]. Further, TC is half as common in black than in white individuals; the ranking of the incidence rate of TC nowadays is as follows, starting with the highest prevalence in white populations, followed by Asians/Pacific Islanders, American Indian/Alaskan natives, and black individuals [13]. Generally, TC occurrence is higher in non-Hispanic males and females than in Hispanic males and females [13]. TC incidence increased by over 200% from 1992 to 2018, while the mortality rate continually remained unchanged [14]. The incidence rate of TC increased in the majority of countries; this increase was the most significant in younger individuals, but generally, rates increased in all individual groups, irrespective of age [15]. The epidemiological landscapes based on the GLOBOCAN database indicate that the mortality rate of TC is less than 1 per 100,000 in most of the investigated countries for both genders; South Korea was indicated to have the highest incidence-to-mortality-rate ratio in both sexes [16]. It was also noted that there are no significant differences between the mortality rates in high-HDI countries and low- and medium-HDI countries [16].

## 3. Classification of Thyroid Cancer

### 3.1. Papillary Thyroid Cancer

Papillary thyroid cancer (papillary thyroid carcinoma, PTC) is the most common type of TC, accounting for nearly 90% of all TC cases [17,18]. Even though PTC is rare in children, it remains the most common pediatric thyroid malignancy, accounting for about 90% of TC in children [19]. Although it is the most prevalent type of TC and might occur in any age group, the peak of its most probable incidence is observed to be between 30 and 50 years of age [20]. The prognosis and the clinical outcome of PTC correlate with tumor size; it was observed that carcinomas that are less than 1.5 cm have a much better prognosis. PTC is observed to be a relatively mild cancer that can be easily operated on; however, in some cases, this type of carcinoma might present significant aggressive behavior associated with such factors as the induction of epithelial–mesenchymal transition, epigenetic modifications, or tumor cell metabolic programming leading to a higher probability of distant metastases and postoperative recurrence [17,21]. There are several aggressive variants of PTC, including the tall cell variant (TCV), diffuse sclerosing variant (DSV), columnar cell variant (CCV), hobnail variant (HV), and solid variant (SV) [22]. PTC usually spreads to the lymph nodes of the neck (primarily to the pretracheal and paratracheal lymph nodes), and the frequency of this type of metastasis is observed in approximately 70% of patients [23]. However, it must be considered that even though cancerous spread via lymph nodes might be associated with a worse recovery and higher risk of recurrence, in the case of PTC, the presence of lymph node metastasis is not associated with a higher mortality rate [24,25]. Only 10% of patients with PTC are estimated to present metastasis at the initial presentation of this malignancy [26]. Distant metastases are not common in PTC, but when they occur, the most common sites include the lungs, bones, liver, and brain [27]. There are several factors associated with a poor prognosis of PTC, but the most prevalent ones include older age at diagnosis (patients > 55 years old), distant metastases and extrathyroidal growth, large tumor size, male sex, aggressive subtypes of PTC, presence of an aneuploid cell population, vascular invasion, and solid and less differentiated areas [28,29]. The overall survival rate of PTC is very high irrespective of the age group; it was estimated that the cure rate might even reach 100% [30]. Similarly, in the case of pediatric patients, the mortality rate associated with PTC is estimated as low to even absent [19].

### 3.2. Follicular Thyroid Cancer

Follicular thyroid cancer (FTC) is the second most common malignancy of the thyroid gland, accounting for about 20% of all cases of TC [31]. It is estimated that FTC accounts for approximately 10% of thyroid malignancies in iodine-sufficient areas while accounting for about 25–40% in areas characterized by iodine deficiency [32,33]. This type of cancer, similar to PTC, more often occurs in females, with a female-to-male ratio equal to 3:1; the mean age at diagnosis is estimated to be 60 years old, while the peak onset age ranges from 40 to 60 years old [34,35,36]. It is estimated that about 80% of FTC cases present mild symptoms with a good prognosis, while the remaining 20% are characterized by aggressive behavior [31]. It was observed that the prognosis of FTC is associated with the size of the tumor, with carcinomas less than 1.5 cm indicating a good prognosis [37]. FTC can be divided into three subtypes, including minimally invasive, encapsulated angioinvasive, and widely invasive variants [38]. Previously, there was another subtype of FTC distinguished, namely Hurthle cell carcinoma; however, the World Health Organization has classified it as a different type of thyroid malignancy [38]. Cases of minimally invasive FTC are more often diagnosed in younger patients; however, it must be taken into consideration that this variant has also been observed to be a probable precursor of more aggressive variants [39]. Invasive follicular carcinoma is primarily characterized by vascular invasion and extension beyond the thyroid gland [40,41]. FTC is characterized by slower progression compared to other thyroid malignancies [42,43,44,45]. It was estimated that about 20% of nonfunctioning follicular adenomas might possess oncogenic mutations that can ultimately lead to the onset of FTC [46]. However, it must be taken into consideration that FTC cannot be distinguished from follicular thyroid adenoma based only on cytologic features [47]. FTC is more aggressive than PTC and tends to metastasize significantly more often; even though distant metastases are not that common for FTC, they are more often observed in this type of carcinoma than in PTC [48,49,50]. Approximately 6–20% of patients with FTC may present distant metastases, which are most often located in the lungs, followed by the bones, liver, brain, and skin [51,52]. The probability of metastases in the case of FTC is greater for older patients [49]. While metastasis to the lymph nodes is not common, vascular invasion is characteristic of FTC [53]. It was estimated that fewer than 10% of patients present metastases into the lymph nodes [54,55].

### 3.3. Medullary Thyroid Cancer

Medullary thyroid cancer (MTC) is a relatively rare neuroendocrine cancer originating from the thyroid parafollicular C cells responsible for calcitonin production [56]. This malignancy accounts for about 1–5% of all TC cases and may appear spontaneously or as a part of several hereditary syndromes, including multiple endocrine neoplasia (MEN) types 2A and 2B and familial medullary thyroid cancer (FMTC) [57]. Among all MTCs, the majority are sporadic, while about 25% account for those associated with MEN2 syndrome [58]. Sporadic MTC has been observed to be more prevalent in females, while the hereditary type of MTC is equally prevalent independently of gender [59]. It is estimated that MTC is the least common among all TCs, accounting for <5% of all thyroid malignancies [60]. The age peak for spontaneous MTC is between 40 and 60 years, with a mean age of 50 years; other trends are observed in cases of MTC associated with genetic diseases [61]. MTC associated with either MEN2A or MEN2B may appear in early childhood, while this malignancy usually occurs in the second to fourth decade when it is a component of FMTC [62,63,64,65,66,67]. In cases when a patient has a family history of MTC and/or MEN2 and presents positive for the *RET* gene mutation, prophylactic surgery may be performed to prevent the onset of MTC [68]. The prognosis of MTC depends on several factors, including the patient’s age, surgical resection status, and the histologic grade of the carcinoma. Generally, older patients presenting high-grade lesions with incomplete surgical treatment present significantly worse prognoses [69]. MTC is characterized by a rather poor prognosis primarily because of its high probability of metastases compared to other thyroid malignancies, as well as delayed diagnosis [57]. Further, high-grade tumors are characterized by poorer overall survival rates compared to low-grade carcinomas because of a higher incidence rate of distant metastases and a higher risk of local recurrence. In addition to metastases in the lymph nodes, distant metastases of medullary thyroid carcinoma can occur in the lungs, liver, or bones, as well as the brain [70].

### 3.4. Anaplastic Thyroid Cancer

Anaplastic thyroid carcinoma (ATC) is a very rare but also very aggressive form of undifferentiated thyroid cancer. This thyroid malignancy accounts for approximately 2% of all TCs [71]. Even though it is very infrequent, ATC accounts for approximately 50% of deaths associated with thyroid malignancies [72]. ATC is more often diagnosed in females; this type of cancer also usually occurs among people of the age over 65 years [73,74]. The etiopathology of ATC is still not fully explained. It is hypothesized that since most of these cancers occur in the setting of a long-standing goiter, its onset might be associated with an undiagnosed differentiated TC [75,76]. It is estimated that about 20–50% of patients with ATC present metastases to distant parts of the body, and the most common sites include the lungs, bones, and brain [77,78,79]. The average survival rate of patients diagnosed with ATC is about five to six months after diagnosis; fewer than 20% of patients are alive one year after diagnosis [80,81]. Factors that might facilitate a better prognosis include a younger age of the patient, tumor size less than 6 cm, unilateral tumor, and lack of lymph node and distant metastases [82].

## 4. Risk Factors and Causes of Thyroid Cancer

**Figure 1:**
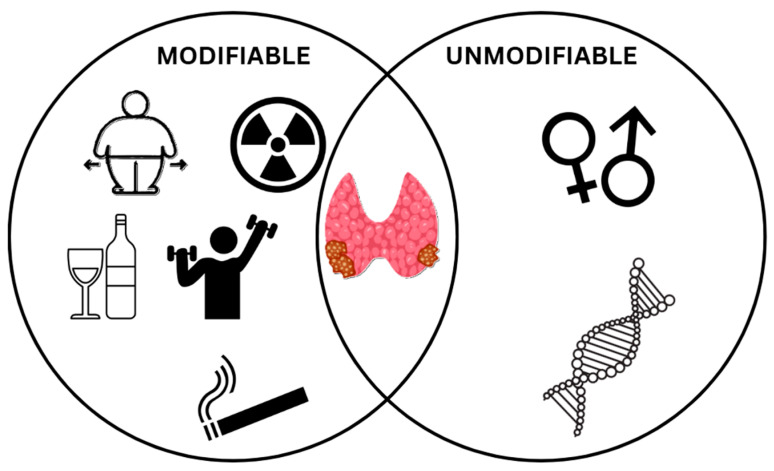
Modifiable and unmodifiable factors in developing thyroid cancer.

TC, being one of the most common malignancies, can be caused by multiple factors. These can be divided into modifiable and unmodifiable [83]. Among modifiable factors, we can distinguish obesity, smoking and secondhand smoking (SHS), heavy alcohol consumption, lack of exercise, and exposure to high levels of radiation. Within the unmodifiable factors, we can include sex, genetic factors such as gene mutations or inherited genetic syndromes, and preexisting benign thyroid disease (Figure 1) [84].

### 4.1. Modifiable Factors

#### 4.1.1. Obesity

**Figure 2:**
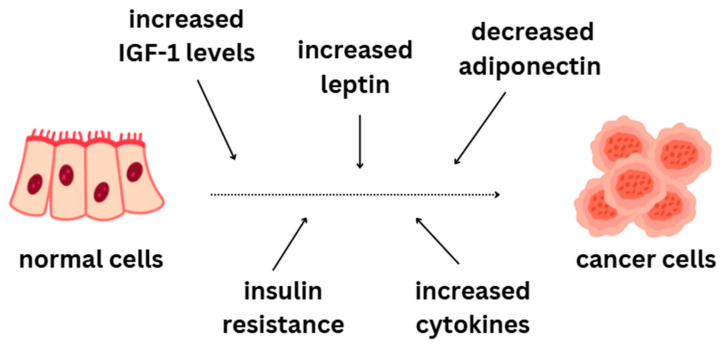
Molecular obesity mechanisms associated with thyroid cancer.

Obesity is an unquestionable risk factor for many diseases, including cancers. It can be highlighted as one of the primary TC causes [85,86]. Regardless of sex, a body mass index (BMI) of 25.0–29.9 is associated with an increased risk of thyroid cancer compared to that for a lower BMI. Obesity may contribute to thyroid cancer through multiple mechanisms, including chronic inflammation, oxidative stress, immune dysfunction, elevated thyroid-stimulating hormone (TSH), insulin resistance, adipokines, and increased aromatase activity. Persistent low-grade inflammation can generate reactive oxygen species, accelerate cell division, and impair tumor suppression. Higher TSH levels may encourage thyroid cell proliferation, genetic mutations, and cancer formation. Insulin resistance leads to increased insulin-like growth factor 1 (IGF-1), which activates cancer-promoting pathways like AKT/mTOR/PI3K and ERK/RAS/MAPK, supporting tumor growth and survival. Additionally, adipokines such as adiponectin, leptin, and resistin may play a role in thyroid carcinogenesis [87]. A 2018 study on obese mouse models showed a link between the overactivation of the JAK/STAT3 signaling pathway and lymphatic metastasis of TC. Obesity affects adipokine levels by increasing leptin and reducing adiponectin. Leptin stimulates cancer cell growth through JAK2/STAT3 and MAPK signaling, whereas adiponectin counteracts tumor progression by blocking the PI3K/AKT/mTOR pathway via AMPK activation. Lower adiponectin levels in obesity may play a key role in TC development and progression [88]. Chronic inflammation activates the transcription of nuclear factor kappa-light-chain-enhancer of activated B (NF-kB), STAT3, and activator protein 1 (AP1), which, cooperatively with hypoxia, promote cancer cell growth and angiogenesis. Tumors contain many immune cells such as tumor-associated lymphocytes, tumor-associated macrophages (TAMs), immature dendritic cells, mast cells, and myeloid-derived suppressor cells. The presence of mast cells and macrophages in tumors is linked to poor TC prognosis. Proinflammatory cytokines produced by NF-kB transcription play a crucial role in cancer progression. The BRAF V600E mutation and RET/PTC gene rearrangement enhance NF-kB activity, increase inflammatory mediator expression, and contribute to lymph node metastases in PTC (Figure 2) [89].

#### 4.1.2. Smoking and Secondhand Smoking

Cigarette smoking and SHS are established risk factors for many diseases and have been implicated in TC. Even minimal SHS exposure induces immediate inflammatory responses, lasting for hours [90]. SHS contains toxic compounds such as thiocyanate, which disrupts thyroid hormone synthesis, and proinflammatory cytokines, for instance IL-1β and IL-6, which may exacerbate thyroid autoimmunity. Additionally, cigarette smoke delivers carcinogens like PAHs and nitrosamines into the bloodstream, leading to chronic inflammation, DNA damage, and potential malignant transformation in thyroid cells [83]. However, emerging evidence challenges the traditional view, suggesting that smoking may exert a protective effect on TC [91]. This protective effect is partly explained by the smoking-induced suppression of TSH, which normally promotes thyroid cell proliferation. Studies indicate that smoking lowers TSH levels while increasing free triiodothyronine (FT3) and free thyroxine (FT4). Conversely, TSH levels tend to rise after smoking cessation Additionally, smoking influences estrogen metabolism and activates the aryl hydrocarbon receptor (AHR) pathway, potentially reducing estrogen’s stimulatory effects on the thyroid. Another proposed mechanism includes increased sympathetic nervous system activity, leading to higher TSH levels [92,93,94,95]. What is more, heavy smokers who quit have a higher likelihood of developing TC than consistent smokers [96]. These conflicting findings highlight the complexity of the smoking–TC relationship and indicate the need for further research.

#### 4.1.3. Alcohol Consumption

Despite popular beliefs, recent studies show that alcohol consumption is negatively correlated with the occurrence of TC [97]. Light to heavy alcohol consumption, when combined with smoking and SHS, is noted to have a submultiplicative effect on TC [95]. Potential mechanisms underlying alcohol’s protective role include alterations in thyroid hormone metabolism, disruptions in thyroid gland function, and impairment of the regulation of the hypothalamic–pituitary–thyroid axis [98]. Regular alcohol intake has been linked to lower TSH levels. Surprisingly, abstinence may increase the probability of developing TC [99]. Meta-analyses estimate that regular alcohol consumption may be associated with up to a 10% lower risk of TC [92]. Nonetheless, the toxic effects of alcohol on the thyroid and their broader systematic health risks must be taken into cautious consideration when interpreting these findings.

#### 4.1.4. Lack of Exercise

There is strong scientific evidence that both physical inactivity and obesity independently elevate the risk of various cancers, whereas regular activity successfully reduces the risk of developing cancer [96]. Recent studies have highlighted the potential role of long non-coding RNAs (lncRNAs) as crucial molecular mediators linking physical activity to cancer prevention. Regular exercise has been shown to modulate the expression of multiple lncRNAs, which are typically upregulated in cancer. These lncRNAs participate in key cellular functions, including epigenetic modifications, cell proliferation, and apoptosis regulation, forming a complex network that may contribute to cancer protection. Abnormal expression of lncRNAs has been strongly associated with various diseases, including cancer, where they contribute to tumor progression, metastasis, and changes in the tumor microenvironment (TME). Emerging research suggests that both exercise and cancer can influence lncRNA levels, highlighting their potential role in disease regulation [100]. Regular exercise regulates the inflammatory response, lowering inflammatory markers like C-reactive protein (CRP), tumor necrosis factor-alpha, and interleukins such as IL-6. Additionally, exercise promotes an anti-inflammatory state by boosting the production of cytokines like IL-1ra and IL-10, which contribute to overall immune balance and disease prevention [101]. Another mechanism highlighting the importance of regular physical activity is the lowering of IGF1 levels, which helps regulate cell growth and prevents cancer progression. Exercise also boosts antioxidant enzyme production, strengthening the body’s defense against oxidative damage with adequate nutrient intake [91]. Furthermore, regular physical activity modulates key processes involved in tumorigenesis, such as proliferation, angiogenesis, and metastasis [96,102]. In TC patients, postoperative exercise significantly reduces the risk of bone fractures, a common complication following thyroidectomy [103]. Overall, physical activity offers many benefits for health and enhances the quality of life in patients being treated for TC [104].

#### 4.1.5. Exposure to High Levels of Radiation

Historically, exposure to high levels of radiation was limited to specific populations. Today, the widespread use of mobile phones has introduced a new, persistent source of radiofrequency (RF) radiation. Emerging studies associate RF radiation from cell phones with an increased risk of TC [105,106]. RF exposure can alter gene-encoded proteins such as PAK6, MDM2, HDAC4, and DACT2, which are involved in tumor growth, progression, suppression, transcription, and apoptosis [105]. However, despite a better understanding of the process behind radiation-induced TC development, there are still no specific markers recognized. Similarities in histological types, oncogenic drivers, and gene expression profiles between radiation-induced and sporadic thyroid tumors suggest that they do not differ from typical markers. With the thyroid gland being highly sensitive to radiation, research has shown that radiation exposure in the head, neck, and chest increases the lifelong risk of developing thyroid malignancies. Radiation therapy in these areas can disrupt thyroid function, often leading to hypothyroidism, which in turn may contribute to cancer development [107]. With the rise in radiological procedures, particularly among pediatric patients, current research is shifting toward assessing risks associated with low-dose radiation exposure [108]. Recent genomic studies on Chernobyl-related TCs have revealed a dose-dependent rise in small deletions and structural variants, likely resulting from DNA double-strand breaks [108,109]. These enable PTC development after radiation exposure [109].

### 4.2. Unmodifiable Factors

#### 4.2.1. Sex

Thyroid cancer incidence is significantly higher in women than in men, a difference thought to be related to reproductive activity and estrogen exposure. Throughout the menstrual cycle, fluctuations in estrogen levels affect circulating TSH concentrations by modulating thyroxine-binding globulin (TBG) levels, altering free thyroxine availability, and stimulating TSH secretion. Estrogens influence TC pathogenesis by promoting cell proliferation, inducing DNA damage, and modulating stress responses. The presence of estrogen receptors (ERα and ERβ) in thyroid tissue, along with the local conversion of androgens into estrogens, suggests direct hormonal involvement in tumor biology. ERα tends to promote thyroid tumor growth, while ERβ inhibits it. Additionally, estrogen-induced DNA adducts and changes in autophagy regulation contribute to tumor progression. Men with TC tend to have worse outcomes than women. This is partly due to later diagnosis, older age at presentation, and more advanced disease stages. However, male sex remains an independent risk factor, especially in patients with BRAF mutations [110]. A 2020 study reported that men with differentiated thyroid cancer (DTC) have a higher risk of recurrence than women. The risk was over three times higher in men with early stage disease but showed no significant difference in advanced stages. Despite receiving more aggressive treatment, men still had higher recurrence rates [111].

#### 4.2.2. Genetic Factors

A 2021 population-based study shows global trends in TC incidence. It reveals various patterns among different subtypes. PTC has seen the most significant rise, especially in high-income and transitioning countries, mainly due to increased detection through advanced imaging. Other subtypes, including FTCs and MTCs, have remained relatively stable without clear temporal trends. The rarest and most aggressive form, ATC, has fortunately shown declining incidence rates in most regions. Overdiagnosis, particularly of PTC, has been a major factor in these trends, leading to calls for more cautious screening and diagnosis approaches [112].

### 4.3. Genes

#### 4.3.1. Papillary Thyroid Cancer

PTC is the most common thyroid malignancy worldwide, regardless of age. Its diagnostic criteria have evolved over the years. Radiation exposure is a well-established risk factor, alongside environmental factors like obesity [113]. PTC is primarily driven by mutations that lead to the constitutive activation of the MAP kinase (MAPK) signaling pathway. These mutations occur in key oncogenes such as BRAF, RAS, RET, and NTRK, which function as upstream regulators of the pathway. The activation of MAPK effectors plays a critical role in tumor initiation, progression, and maintenance [114]. Although PTC generally has a favorable prognosis with a >90% 10-year survival rate, recurrence (especially in neck lymph nodes) occurs in 20–30% of cases. Early diagnosis is crucial, as traditional FNA cytology has limitations. A valuable biomarker, BRAF V600E mutation, may be crucial for PTC identification [115]. PTC can be hereditary, and some of these cases are linked to known syndromes such as Cowden syndrome, Gardner syndrome, and the Carney complex, where specific genetic mutations have been identified [116]. BRAF V600E is the most frequent mutation, resulting from a T-to-A transversion at nucleotide 1799. This mutation leads to the continuous activation of BRAF kinase, which subsequently phosphorylates MEK and ERK, driving uncontrolled cell proliferation. It is associated with aggressive tumor features, extrathyroidal invasion, and poorer prognosis. Additionally, AKAP9–BRAF fusion, caused by chromosomal inversion, has been identified in radiation-induced PTC [114]. RAS mutations (in NRAS, HRAS, and KRAS) occur in about 10–20% of cases and are more frequently associated with the follicular variant of PTC [114,117]. Additionally, RET/PTC rearrangements, resulting from chromosomal translocations, are present in 10–20% of cases. RET/PTC1 and RET/PTC3 mutations are the most common variants, particularly in radiation-induced pediatric PTC cases [114,117,118]. These mutations play a crucial role in tumor initiation and progression, influencing prognosis and potential therapeutic targets (Table 1) [117].

#### 4.3.2. Medullary Thyroid Cancer

MTC is a rare neuroendocrine tumor, accounting for 3–5% of thyroid malignancies. Despite its low incidence, MTC is responsible for a significant proportion of TC-related deaths. It can occur sporadically or as part of hereditary syndromes like multiple endocrine neoplasia type 2 (MEN2) and FMTC. Calcitonin and carcinoembryonic antigen (CEA) serve as crucial diagnostic and prognostic markers for MTC [119,120]. Mutations in the RET proto-oncogene play a crucial role in the development of MTC and are classified into germline and somatic mutations. The RET gene encodes a receptor tyrosine kinase involved in cell proliferation, differentiation, and survival. Mutations can affect different RET domains, leading to distinct clinical outcomes. Extracellular RET mutations, especially in codon 634, are commonly linked to MEN2A syndrome, often resulting in early onset MTC. Codon 918 mutations are strongly associated with MEN2B syndrome, which is characterized by early and aggressive MTC, often with metastases in infancy. Intracellular mutations are usually seen in FMTC and result in milder disease progression. Genetic testing for RET mutations is recommended for all MTC patients to guide risk assessment, early diagnosis, and prophylactic thyroidectomy in at-risk individuals [119]. About 70% of RET wild-type sporadic MTCs harbor RAS mutations, with HRAS being the most common, followed by KRAS and rare NRAS mutations. RET and RAS mutations are usually mutually exclusive, though rare cases with both mutations exist. Within the other molecular alterations, we can distinguish CDKN2C loss, found in 20% of tumors and associated with distant metastases and reduced survival, and mTOR pathway activation, linked to lymph node metastases and more prevalent in RAS-mutant MTC. Increased EZH2 and SMYD3 expression are observed in aggressive tumors. MiRNA overexpression is linked to metastasis and worse outcomes [121]. A 2025 case report shows a possibility of a connection between a germline pathogenic variant in the CDKN1B gene [multiple endocrine neoplasia type 4 (MEN4)] and MTC. This case highlights the coexistence of MTC and MEN4 linked to a novel CDKN1B germline frameshift mutation, expanding the understanding of MEN4-associated endocrine tumors [122].

#### 4.3.3. Follicular Thyroid Cancer

FTC is the second most prevalent type of differentiated TC. It originates from follicular cells, which are responsible for producing and secreting thyroid hormones. FTC is more common in women and typically presents during the fifth and sixth decades of life. Risk factors for FTC include iodine deficiency, age over 50 years, and female sex [34]. FTC is characterized by several genetic mutations that play pivotal roles in its pathogenesis. Mutations in the RAS family of oncogenes—specifically NRAS, HRAS, and KRAS—are among the most prevalent, detected in approximately half of FTC cases. They have been associated with poor prognosis and distant metastases. These mutations often occur at codon 61 and lead to the constitutive activation of signaling pathways that drive tumor development. Additionally, TERT promoter mutations are found in about 25% of FTCs and are linked to aggressive clinical outcomes, including advanced TNM stage, recurrence, and higher mortality rates. The PAX8-PPARγ fusion gene is present in approximately one-third of FTC cases and contributes to tumorigenesis through altered transcriptional regulation [123,124]. Furthermore, mutations in the EIF1AX gene have been identified in a subset of FTCs, often co-occurring with RAS mutations, suggesting a collaborative role in cancer progression [125]. Other mutations, such as EZH1, have been identified in follicular-patterned lesions, typically in lower-grade tumors [124].

#### 4.3.4. Anaplastic Thyroid Cancer

ATC, representing 5–10% of TCs, is an extremely aggressive tumor with low survival rates. Distant metastases are present at the time of diagnosis in 30–40% of cases. Under microscopic examination, it exhibits a diverse cellular structure and a complete loss of follicular differentiation. ATC is frequently linked to coexisting DTC or a prior history of DTC, which suggests that certain DTC clones may undergo morphological changes leading to dedifferentiation. ATCs exhibit significantly higher frequencies of TP53, TERT promoter, PIK3CA, and PTEN mutations. Additionally, ATCs carry mutations in ATM, NF1, NF2, CDKN2A, CDKN2B, and RB1 [123,126,127]. ATCs frequently harbor TP53 mutations, disrupting tumor suppression and enabling uncontrolled growth. TERT promoter mutations, often co-occurring with BRAF or RAS alterations, enhance telomerase activity for indefinite proliferation. Disruptions in cell cycle regulators such as CDKN2A, CDKN2B, and RB1 further drive unregulated division. The PI3K/AKT/mTOR pathway is commonly activated via PIK3CA and PTEN mutations, promoting survival and metabolic reprogramming. DNA repair deficiencies, including ATM and NF1/2 mutations, increase genomic instability. Epigenetic regulators like ARID1A, SMARCA4, and histone-modifying enzymes are frequently mutated, contributing to aggressive phenotypes. Structural alterations, such as CNVs, impact tumor suppression. EIF1AX mutations, often with RAS alterations, may dysregulate protein synthesis. TAMs create an immunosuppressive microenvironment, further supporting tumor progression [127].

#### 4.3.5. Preexisting Benign Thyroid Disease

Individuals with a history of benign thyroid diseases, including hyperthyroidism and goiter, are at an increased risk of developing TC. This association is likely mediated by chronic alterations in TSH levels, which may promote abnormal thyroid cell proliferation Additionally, studies have shown that a family history of benign thyroid conditions further raises the likelihood of TC, indicating a potential genetic link [107]. Patients with preexisting thyroid-specific autoimmune diseases, such as Hashimoto thyroiditis (HT) and Graves’ disease, have a significantly increased risk of developing TC [128,129]. A 2021 study showed a connection between HT and an increased risk of developing thyroid malignancies, especially PTC and MTC. Several mechanisms have been proposed to explain this association. One mentions the inflammatory process in HT resulting in DNA damage and further mutations. Another highlights the possible thyroid tissue proliferation caused by the constant increase in TSH levels. However, specific mechanisms are still unknown [130]. Interestingly, thyroid cancers arising in patients with preexisting autoimmune thyroid disease tend to present as smaller tumors with lower rates of lymph node metastasis. This observation suggests that immune-mediated factors may not only increase cancer risk but also modulate tumor behavior. Chronic inflammation, oxidative stress, and dysregulated TSH signaling are regarded as key contributors to thyroid carcinogenesis in this context (Table 2) [129].

## 5. Symptoms and Diagnosis of Thyroid Cancer

Thyroid cancer (TC) can present a wide range of symptoms. Research on the distress, anxiety, and depression experienced by individuals with advanced TC shows that over half of the participants experience various symptoms, with sleep disturbances, fatigue, and emotional distress being the most prevalent and severe. Moreover, symptoms like hoarseness, numbness, and a noticeable lump in the thyroid region are often observed, typically due to tumor interaction with the recurrent laryngeal nerve and parathyroid gland. Over 30% of patients report symptoms of anxiety and depression [131]. However, in many cases, an enlarged thyroid, which may be structurally abnormal, is diagnosed incidentally—often following an X-ray of the chest or neck conducted for reasons unrelated to thyroid malignancies. Approximately 50% of thyroid cancer cases are discovered incidentally, particularly in asymptomatic patients. These often involve small papillary thyroid cancers (PTCs). Additionally, TC is frequently identified histologically when thyroid glands are removed for benign conditions, revealing small tumors incidentally [132]. The diagnosis of TC typically involves a combination of physical examination, imaging studies (e.g., ultrasound), and fine-needle aspiration biopsy (FNAB). Further tests, including molecular markers and radioactive iodine scans, are used to determine the cancer’s type and extent [133]. The abnormal and continuous growth of cancerous cells within the thyroid leads to gland enlargement, which can be detected during regular check-ups. The enlarged thyroid may be unrecognized by the patient if the growth is minor and asymptomatic, or it may be easily identified if it causes discomfort or difficulty in swallowing [134]. The increase in the number of malignant cells can manifest as an enlarged gland or the presence of detectable nodules, which can be felt during a physical examination. Alarm symptoms identified during palpation often lead to further diagnostic testing, including serum TSH levels and neck ultrasound. Alarming ultrasound features that may prompt a biopsy include microcalcifications, neovascularization, disorganized internal vascularity, irregular margins, and a taller-than-wide shape [135]. Thyroid nodules, though often causing concern, are malignant in only a small percentage of cases, especially in adults. Ultrasonography (US) and fine-needle aspiration cytology (FNAC) remain the primary methods for assessing malignancy risk. Advances in US technology, such as elastography, have improved diagnostic accuracy, particularly for indeterminate nodules [136]. More recently, the introduction of molecular testing for genetic mutations has significantly enhanced the diagnosis of various thyroid malignancies and facilitated targeted therapy [137]. Molecular tests provide additional diagnostic accuracy beyond cytology alone. The Bethesda System for Reporting Thyroid Cytopathology (TBSRTC) classifies thyroid nodules into six categories based on cytologic evaluation. Molecular testing has been especially useful for Bethesda III and IV nodules, where cytology’s indeterminate nature presents a challenge in clinical decision-making. These tests can identify gene mutations and alterations correlated with varying malignancy risks. Aggressive TC is associated with BRAF V600E, RET/PTC, and TERT promoter mutations, whereas NRAS, HRAS, KRAS, BRAF K601E, and PAX8/PPARG alterations are more commonly linked to benign nodules, non-invasive follicular thyroid neoplasms with papillary-like nuclear features (NIFTPs), or low-risk differentiated thyroid cancers (DTCs). Preoperative molecular testing can optimize surgical decisions, particularly for Bethesda V and VI nodules, when compared to cases without molecular testing [123,138,139,140]. Both RNA and DNA–RNA molecular tests can be used to diagnose malignancy in indeterminate thyroid nodules, with no significant difference between the two. Both demonstrate high sensitivity and reasonable specificity [141]. Ultrasonography (US) remains the primary method for evaluating thyroid lesions. If malignancy is suspected or the ultrasound results are inconclusive, single-photon emission computed tomography (SPECT) or ultrasound-guided fine-needle aspiration (FNA) may be used for follow-up. Computer tomography (CT) is typically employed to assess metastasis, while magnetic resonance imaging (MRI), with its excellent soft-tissue contrast and high resolution, is helpful in distinguishing between benign and malignant nodules. Radioiodine is the preferred diagnostic and therapeutic approach due to the preservation of sodium–iodide symporter (NIS) in well-differentiated thyroid tumors. For poorly differentiated or dedifferentiated TC, PET/CT and PET/MRI are particularly useful, with emerging evidence suggesting that PET/MRI may offer superior staging for TC, both at initial diagnosis and during follow-up evaluations [142,143]. Papillary thyroid cancer (PTC) often lacks clinical manifestations, with neither hyperthyroidism nor hypothyroidism typically present, as PTC rarely affects hormone production. The diagnosis is often made when an enlarged gland is detected, either by the patient or during a clinical examination, typically due to swallowing or breathing difficulties [26,144]. Extrathyroidal growth of PTC occurs in about 10% of patients [145], and distant metastases are an uncommon manifestation of the disease [146,147]. Follicular thyroid cancer (FTC) usually presents as a solitary thyroid nodule, often accompanied by thyroiditis or nodular hyperplasia. Patients may remain asymptomatic for a long time, with symptoms appearing only in cases of significant gland enlargement, potentially leading to dyspnea, tracheal compression, or dysphagia. Currently, it is not possible to differentiate follicular thyroid adenomas from carcinomas based solely on cytomorphologic criteria. However, the use of reverse-transcriptase polymerase chain reaction (PCR) for TSH receptor and thyroglobulin messenger RNA can significantly improve the differentiation between these malignancies. FTC is confirmed through pathological examination showing follicular cells that lack nuclear atypia, with diagnosis primarily based on evidence of capsular and vascular invasion [34]. Medullary thyroid cancer (MTC) manifests similarly to PTC or FTC, presenting as a thyroid nodule with comparable symptoms. However, MTC is characterized by the excessive secretion of calcitonin, which can lead to symptoms such as diarrhea and flushing. Other substances, such as prostaglandins, serotonin, and vasoactive intestinal peptide (VIP), may also contribute to diarrhea in MTC. Serum levels of carcinoembryonic antigen (CEA) should be measured as part of the diagnostic workup for MTC [57,148,149]. Anaplastic thyroid cancer (ATC) typically presents with a rapidly growing mass that compresses surrounding structures, leading to dysphagia and dyspnea. A firm, painful neck lump, often accompanied by swollen lymph nodes, is common in ATC [150]. About half of ATC cases present with metastases at diagnosis and are classified as stage IV tumors. Therefore, it is crucial to conduct ultrasound, CT, MRI, and FNAB to assess tumor extent, local invasion, and possible distant metastases (Table 3) [71]. In recent years, high-throughput sequencing technologies have made major contributions to the molecular characterization of thyroid cancer [151]. Next-generation sequencing (NGS), whole-exome sequencing (WES), and RNA sequencing (RNA-Seq) provide a complete detection of point mutations, gene fusions, and expression profiles across numerous thyroid cancer subtypes [152]. NGS panels that target common changes like BRAF V600E, RAS mutations, RET/PTC rearrangements, and PAX8-PPARy fusions can improve diagnosis accuracy and inform treatment recommendations for intermediate cytology cases (Bethesda III/IV) [153]. RNA-Seq also aids in the discovery of uncommon fusion transcripts and gene expression profiles [154]. These technologies are progressively being integrated into clinical processes, led by ATA and NCCN recommendations, especially when conventional procedures produce unclear results [155].

## 6. Diagnostic Markers of Thyroid Cancer

Diagnostic indicators are critical in the diagnosis of TC because they allow for early detection of the disease, which is necessary for effective treatment and a better prognosis. The identification of relevant indicators enables exact diagnosis, which influences treatment selection and progress tracking.

### 6.1. Thyroglobulin

More than 90% of DTCs are PTCs, and thyroglobulin (TG), a key substrate for thyroid hormone production, is an important tumor marker for DTC [157]. Normally, differentiated thyroid cells produce TG [158], which is a 330 kD, 2750-amino-acid protein produced by the follicular epithelial cells of the thyroid [159]. These cells are found primarily in thyroid follicular cells and follicular lumens [159]. Autoantibodies to the TSH receptor can elevate TG levels, which can result in higher circulating levels in thyroid cancers that begin in follicular epithelial cells [160]. TG emphasizes the need for close monitoring and is used to identify recurrent disease and track residual disease [158]. Preoperative serum TG levels have the potential to differentiate between benign and malignant thyroid nodules in patients with equivocal cytology [161]. Validated immunoassays calibrated against approved reference materials should be used to measure serum TG levels. Laboratories performing TG tests must adhere to recognized national or international quality assurance programs [162]. Tg secretion is known to occur in both benign and well-differentiated malignant thyroid tissue [163]. In fact, the presence of anti-TG antibodies (TgAb) may affect TG test results, and the presence of normal thyroid remnants reduces the utility of TG [164]. According to a study, there is a nonlinear correlation between preoperative TG levels above 1.39 ng/mL and a significant increase in the risk of distant metastases in PCT [165]. In patients with diabetes and TC, serum TG levels may be associated with HbA1c levels; this association is stronger in individuals with fluctuating TG levels, which requires further study [166]. This association highlights the need for close monitoring and elucidating the complex links between TC and other metabolic disorders.

### 6.2. Calcitonin

MTC is an aggressive neuroendocrine tumor that releases the neurotransmitter calcitonin gene-related peptide (CGRP) and develops from thyroid parafollicular cells, usually with a poor prognosis [167]. CGRP expression in MTC is linked to aberrant dendritic cell (DC) formation [167]. Calcitonin (Ctn) secretion is recognized as one of the primary features of MTC [57]. Slight elevations in Ctn may be observed in individuals with C-cell hyperplasia, autoimmune thyroiditis, chronic renal illness, hyperparathyroidism, and some lung and neuroendocrine tumors, potentially leading to false-positive or false-negative results [168]. Ctn and CEA have been proposed as biochemical indicators for MTC according to the National Comprehensive Cancer Network and American Thyroid Association (ATA) Guidelines for Management of MTC [169]. Human plasma includes one or more Ctn-degrading enzymes, and serum proteases digest the hormone more quickly in plasma [170]. A drop in Ctn levels of less than 50% 30 min following thyroidectomy and central neck lymph node dissection (LND) suggests that tumor tissue persists in MTC patients [171]. With a 10-year survival rate of 97.7%, long-term postoperative Ctn normalization as a biochemical cure is a favorable prognostic factor associated with a better outcome [172]. Serial Ctn tests may be more sensitive than radiological follow-up in advanced MTC [173]. Serum Ctn levels are a reliable and accurate biochemical predictor of tumor development for postoperative monitoring [174]. In addition, higher Ctn levels may play an important role in the early diagnosis of MTC, especially in patients with a family history of medullary thyroid cancer [175,176]. Therefore, Ctn serves as both a diagnostic and prognostic biomarker, improving the precision of treatment and follow-up strategies in patients with MTC.

### 6.3. Carcinoembryonic Antigen

CEA is a glycoprotein initially identified as being involved in intercellular adhesion. It is expressed by neuroendocrine tissues of the gastrointestinal tract during fetal development [177]. CEA is commonly recognized as a marker of various malignant diseases, but it does not show specificity for MTC when used alone. However, its combined use with Ctn significantly increases diagnostic sensitivity for MTC [178]. Of note, serum CEA levels are commonly used to monitor disease progression in patients with MTC [179]. Normal CEA levels in healthy individuals range from 2 to 4 ng/mL, with levels above 10 ng/mL usually associated with malignant conditions [180]. More than 50% of patients with MTC have benign increases in CEA [179,181]. Immunohistochemical studies show that patients with stronger CEA staining have a more aggressive, diffuse subtype of MTC [168]. Interestingly, CEA can be detected in C cells at all stages of MTC progression [181]. The rare nature of MTC should be taken into account when examining CEA elevations [182]. Disease progression can vary considerably over time but can be relatively well estimated by examining the doubling time of Ctn and CEA levels [181]. Furthermore, the risk of central lymph node metastasis is significantly increased when CEA levels exceed 30 ng/mL [183]. Despite its role in MTC, CEA is also involved in various endothelial cell functions, including cell adhesion, proliferation, and migration, both in vivo and in vitro [184]. It is mainly metabolized in the liver, and hepatic or biliary dysfunction can lead to elevated CEA levels, resulting in false-positive results [185]. CEA levels above 271 ng/mL are significant for advanced tumor size and stage, central compartment metastasis, and a decreased likelihood of biochemical cure, whereas levels above 500 ng/mL are associated with significantly higher patient mortality [186]. This highlights the importance of CEA levels as markers for assessing prognosis in MTC. Interestingly, a study found that patients with sporadic MTC had higher postoperative CEA levels and lower trough Ctn levels compared with patients with hereditary forms of MTC [187]. The revised ATA guidelines indicate that CEA should not be considered a specific biomarker for MTC, although it is still useful in disease management. Surgical treatment of patients with elevated CEA levels, particularly those above 30 µg/L, typically includes total thyroidectomy, central cervical lymph node dissection, and unilateral lateral cervical LND [188]. Furthermore, monitoring the doubling time of Ctn and CEA levels after surgery provides sensitive markers for assessing the progression and aggressiveness of metastatic MTC [189].

### 6.4. Procalcitonin

Procalcitonin (Pct) is a 116-amino-acid precursor produced by thyroid C cells and is considered a more reliable and stable biomarker than Ctn for the diagnosis and monitoring of MTC, which is released by parafollicular cells in the thyroid [190]. In addition to its role in MTC, PCT is widely used to monitor inflammatory activity and is a key marker in differential diagnosis, especially in the identification of bacterial infections [191]. It has been recognized as a valuable biomarker for the early detection of systemic bacterial infections, and treatment is usually initiated when Pct levels exceed a threshold of 2 ng/mL [192]. Pct levels do not increase during viral infections, making it a more specific marker for bacterial infections [192]. It is also the most reliable marker for sepsis [193]. In healthy individuals, Pct levels are very low because prohormones are not secreted into the circulation [194]. Elevated Pct levels can be observed in various settings, such as trauma, surgery, heat stroke, immunomodulatory therapy, and malignant diseases, particularly neuroendocrine tumors and metastatic tumors [195]. Pct production is associated with the presence of tumor necrosis factor (TNF) and several inflammatory cytokines, including interleukin-1, interleukin-2, and interleukin-6 [196]. In addition to thyroid C cells, Pct is produced by other tissues in response to severe systemic inflammation, local bacterial infections, autoimmune diseases, trauma, surgery, and fungal or parasitic infections [197]. A major advantage of procalcitonin over other biomarkers is its stability. Pct has a longer half-life, better thermal stability, and standardized cutoff points, making it a valuable tumor marker in the diagnosis and monitoring of MTC, and it also correlates with tumor size and progression in patients with MTC [198]. During the COVID-19 pandemic, persistently high Pct levels have helped uncover previously undiagnosed cases of MTC, highlighting its potential for early detection and treatment [199]. Furthermore, when Pct levels remain elevated after infection, especially in combination with significant CEA levels, it may aid in the early detection of MTC [190]. Pct also plays a key role in calcium homeostasis [200]. However, one limitation is that Pct production is not exclusive to C cells, as it is also produced by neuroendocrine cells in the lung and gut, as well as in other tissues in response to various inflammatory and infectious conditions [191,192,193,201].

### 6.5. Thyroid-Stimulating Hormone

TSH is mainly produced by basophils in the distal part of the adenohypophysis and plays a vital role in thyroid function. It acts on thyroid tissue by binding to TSH receptors (TSHRs), which stimulate the production and release of thyroid hormones, including thyroxine (T4) and triiodothyronine (T3) [202]. Through its receptor, TSH promotes the growth and activity of thyroid follicular cells, influencing the synthesis and secretion of these hormones [202]. TSH also stimulates growth in thyroid follicular cells, resulting in thyroid enlargement [203]. TSH receptors are found on the cell membrane of DTC cells, and their activation encourages cell growth by increasing the expression of thyroid-related proteins such as TG [204]. It is widely recognized that high TSH levels stimulate thyroid cell proliferation, which can lead to lymph node metastasis and invasion of nearby tissues [205]. This characteristic of TSH has led to the implementation of TSH suppression strategies in postoperative patients to reduce tumor growth and minimize recurrence risks [206]. However, excessive TSH suppression, particularly in postmenopausal women, has been linked to negative effects such as osteoporosis and a higher risk of fractures [207]. Research also shows that maintaining serum TSH levels below 2 mU/L does not significantly affect the odds ratio for recurrence [204]. However, when serum TSH reaches 2 mU/L or higher, adverse outcomes, including the recurrence of DTC and cancer-related death, have been observed [208]. A retrospective study from 1977 indicated that TSH suppression treatment could significantly lower recurrence rates [209]. Interestingly, some studies suggest that lower TSH levels might actually increase the risk of thyroid cancer and goiter, a surprising finding since TSH is usually associated with promoting the growth of thyroid cancers [210]. Factors such as gender, BMI, T stage, and preoperative FT4 levels have been identified as independent risk factors that affect the success of TSH suppression [211]. These findings emphasize the complex role of TSH in thyroid cancer biology and the need for careful management of TSH levels in patients.

### 6.6. microRNA

Exosomal microRNAs (miRNAs) have emerged as promising biomarkers, offering significant potential to enhance prognostic assessments in various cancers, including PTC [212]. MiRNAs are small, non-coding RNA molecules, typically ranging from 19 to 25 nucleotides, that function as negative regulators of gene expression by binding to the 3′ untranslated regions of target messenger RNAs (mRNAs) in the cytoplasm [213]. This interaction results in translation repression and mRNA degradation, which profoundly influence gene expression within cells [214]. MiRNAs are known for their stability, making them accessible from a wide range of biological samples, such as blood, tissue biopsies, and even formalin-fixed paraffin-embedded tissues [215]. Furthermore, circulating miRNAs—those released into the bloodstream—serve as reliable indicators of cellular changes, with their levels potentially reflecting clinical features, treatment responses, and patient outcomes [216]. In thyroid cancer, miRNAs have been implicated both as oncogenes and tumor suppressors [217]. Specific miRNAs, like miR-31, have shown conflicting evidence in their relationship with the BRAFV600E mutation in PTC [218]. Other miRNAs, such as miR-129-5p, have demonstrated the ability to suppress the migration, proliferation, and lymph node metastasis (LNM) of PTC cells, although further research is needed to better understand their roles [219]. Additionally, the expression levels of miRNAs such as miR-221, miR-222, and miR-146 have been shown to distinguish between malignant and benign thyroid nodules, marking them as potential diagnostic biomarkers for early cancer detection [220,221]. Moreover, miRNAs like miR-136 are abnormally expressed in metastatic tumors, playing a significant role in tumor development and progression [222,223]. The interplay between miRNAs and genetic mutations in thyroid cancer is also linked to tumor aggressiveness and poorer prognosis [224], highlighting their importance in personalized medicine. Overall, miRNAs are excellent candidates for diagnostic, prognostic, and predictive patient stratification, offering hope for more accurate and less invasive cancer diagnostics [225].

### 6.7. BRAF

It has been demonstrated that the tumor cell proliferation index, measured by Ki67 expression, serves as an effective prognostic marker for MTC [226]. BRAFV600E is the most frequent genetic mutation in DTC, occurring in 60% of patients, and contributes to the development of malignant tumor cell phenotypes, including proliferation, metastasis, and immune escape [218]. This V600E mutation, for instance, reduces the expression of genes responsible for iodine metabolism, thereby diminishing tumors’ responsiveness to iodine-131 (131I) [227]. Papillary carcinoma is more commonly observed in patients with the BRAF mutation than in those without it [228]. In cases where a RAS-like mutation is detected, there is uncertainty regarding the malignancy risk of the nodule [218]. Anaplastic thyroid cancer, the most aggressive form of thyroid cancer, is found to harbor BRAF mutations in over 40% of cases [229]. In normal thyroid tissue, the wild-type BRAF gene is transcribed into the BRAF protein, which is activated by the RAS family [229]. BRAF is a cytoplasmic serine–threonine protein kinase that plays a crucial role in the MAPK signaling pathway. Among the RAF family members, BRAF is the only one activated by mutations in human cancers. However, BRAFV600E should not be used as the sole prognostic marker in low-risk thyroid cancer due to its low positive predictive value [230,231]. In conclusion, BRAF mutations, particularly BRAFV600E, are a significant risk factor for LNM in PTC. Research indicates that in conventional PTC, the presence of the BRAF mutation increases the risk of death related to LNM, a risk that is absent in BRAF wild-type cases [232]. Thyroid nodules with BRAF mutations that are not V600E exhibit a higher malignancy rate (73%), though still lower than that for nodules with BRAFV600E mutations. According to ATA guidelines, most of these nodules are classified as low risk, with only 8% being considered intermediate or high risk [233].

### 6.8. RAS

RAS mutations, particularly in the KRAS proto-oncogene, are crucial in the progression of ATC, a highly aggressive form of thyroid cancer. KRAS is part of a family of small GTP-binding proteins, and mutations in this gene can lead to the constitutive activation of the RAS/MAPK signaling pathway, promoting tumor growth and metastasis [234,235]. These mutations are observed in approximately 13% of PTC cases, where they often maintain some degree of follicular structural differentiation [236]. On the other hand, FTCs are more commonly associated with RAS mutations, placing them in the RAS-like category of thyroid tumors [237,238]. In both FTCs and PTCs, especially those with poorly differentiated areas, RAS mutations have been identified as important diagnostic markers. These mutations can be particularly useful when malignancy is not clearly evident through standard morphological evaluation [239,240]. Moreover, the presence of RAS mutations correlates with a significantly higher risk of distant metastasis and increased mortality, suggesting their potential as a prognostic marker in thyroid cancer [241]. It is also noteworthy that most tumors with RAS mutations are classified as the follicular variant of PTC, further emphasizing the role of these mutations in thyroid tumorigenesis [242]. Collectively, RAS mutations play a pivotal role in the development and progression of thyroid cancer, with important implications for diagnosis and prognosis.

### 6.9. RET

RET mutations play a significant role in the development of thyroid cancer, particularly in PTC [243]. One of the key genetic aberrations implicated in the development of PTC is the RET chromosomal rearrangement, commonly referred to as RET/PTC [244]. This rearrangement is most frequently associated with follicular cell-derived thyroid cancers [245]. RET/PTC fusions are known to result in the formation of a fusion gene, and they are present in more than 70% of radiation-induced thyroid cancers [246]. This highlights the crucial role of RET rearrangements in thyroid carcinogenesis, especially in the context of radiation exposure. The RET proto-oncogene encodes a cell membrane receptor tyrosine kinase, and when altered, it can lead to the activation of oncogenic signaling pathways that drive tumor formation [238,247]. There are various types of RET/PTC rearrangements, defined by different fusion partner genes, with RET/PTC1 and RET/PTC3 being the most common forms [248]. These rearrangements are more frequently found in malignant thyroid tissues than in benign nodules, and they are strongly associated with more aggressive tumor behavior, including a higher likelihood of lymph node metastasis [249]. RET/PTC is particularly prevalent in pediatric thyroid cancers, where it is the most common mutation found in children and adolescents with thyroid cancer [250]. In these patients, RET/PTC mutations are often linked to more aggressive tumor behavior and an increased frequency of metastatic dissemination [251]. Interestingly, benign thyroid lesions that harbor RET/PTC translocations are also characterized by a rapid growth rate, which often necessitates early surgical intervention [252]. This rapid progression underscores the potential malignancy of thyroid lesions harboring RET/PTC rearrangements, even if initially benign [253]. Moreover, while somatic RET mutations are generally considered negative prognostic indicators, some studies argue that these mutations do not necessarily correlate with compromised disease-specific survival (DSS) or overall survival (OS) in patients with MTC [254]. However, it is important to note that children who possess RET codon mutations exhibit the most severe manifestations of MTC, highlighting the potential for worse outcomes in this population [255]. In summary, RET/PTC rearrangements are a crucial molecular event in thyroid cancer, especially in PTC, and are strongly associated with aggressive disease, frequent metastasis, and rapid tumor growth. While the prognostic implications of RET mutations can vary, their role in the pathogenesis of thyroid cancer remains a significant focus of ongoing research [256].

### 6.10. Summary

Among the discussed biomarkers, thyroglobulin (Tg) and calcitonin (Ctn) hold the greatest clinical importance as the primary markers for differentiated thyroid cancer (DTC) and medullary thyroid cancer (MTC), respectively. While both are well-established tools for postoperative monitoring and assessing treatment response, their diagnostic accuracy may be limited by interfering factors such as Tg autoantibodies or comorbid conditions. CEA and procalcitonin (Pct) enhance the prognostic value of Ctn in MTC but lack sufficient specificity to serve as standalone markers. Emerging molecular markers—including microRNAs, BRAF and RAS mutations, and RET/PTC rearrangements—support subtype-specific diagnosis and risk stratification and may guide targeted therapy in the future, though further clinical validation is needed. Importantly, the diagnostic and prognostic relevance of each marker varies significantly across thyroid cancer subtypes, making histological context and individual patient characteristics essential for accurate interpretation (Table 4).

## 7. Current Treatment Strategies

### 7.1. Surgical Treatment of Thyroid Cancer

#### 7.1.1. Papillary Thyroid Cancer

At present, surgery is the most common and effective method for treating PCT [26]. The choice of surgical procedure for PCT depends on the patient’s specific clinical condition and the characteristics of the carcinoma itself. In some situations, the entire thyroid gland must be removed, while in other cases, a thyroid lobectomy is performed to remove only the affected lobe. For patients with unilateral goiter, low baseline Ctn levels, and small tumors (≤2.5 cm), a lobectomy combined with central (±lateral) LND can be an effective alternative to more extensive procedures such as total thyroidectomy [257]. For unilateral tumors, lobectomy along with central (±lateral) LND may be sufficient, but for bilateral or larger (metastatic) tumors, more extensive surgery is required. The decision on the extent of surgery should be based on imaging results (such as ultrasound) and Ct levels [258]. In children with DTC, total thyroidectomy, along with bilateral LND of the central compartment and dissection of affected lateral compartments, is recommended [259]. While PCT generally has a favorable prognosis when diagnosed early, it is often associated with a high rate of LNM. There is no agreement on whether prophylactic LND should be a standard procedure for patients with cN0 PTC, though some research indicates it may improve outcomes and help avoid unnecessary subsequent surgeries, especially when risk factors are considered [260]. However, aggressive LND can lead to a range of postoperative complications, including hypoparathyroidism, chylous leakage, injury to the cervical plexus, recurrent laryngeal nerve damage, and neck numbness, which can all negatively affect the patient’s quality of life [261]. In terms of LNM in PTC, our study supports previous findings that younger age, multifocality, larger tumor size, and extrathyroidal invasion are independent risk factors for LNM in PTC patients [262]. Thyroid nodules are common in the general population, with prevalence rates ranging from 50% to 67% in autopsy studies [263]. Ultrasound imaging of thyroid nodules, followed by fine-needle aspiration biopsy for cytological analysis, remains the gold standard for pre-surgical diagnosis [264]. Additionally, a study showed that total thyroidectomy (TT) did not offer better therapeutic outcomes than thyroid lobectomy in low-risk DTC patients. Consequently, thyroid lobectomy was included as a treatment option in the 2015 ATA guidelines, which has led to an increase in both the number of low-risk DTC patients and the number of those undergoing thyroid lobectomy [204].

#### 7.1.2. Follicular Thyroid Cancer

FTC often develops without noticeable symptoms in its early stages, making it challenging to detect early. The standard treatment for FTC is thyroid lobectomy, but this approach is not suitable for larger tumors (>4 cm) or those with significant extrathyroidal extension [265]. Additionally, surgical options are not recommended for patients with aggressive mutations associated with FTC [266]. In some cases, surgery might be considered for nodules that are initially classified as benign based on cytology and/or low risk on ultrasound but later become symptomatic [267]. However, for nodules with indeterminate cytology (Bethesda class III and IV), where active surveillance is not appropriate—such as in cases of large size, high suspicion of malignancy on ultrasound, or the presence of symptoms—surgical intervention may be necessary [268]. For minimally invasive FTC, the typical treatment involves thyroid lobectomy and isthmectomy, while for invasive FTC, the preferred treatment includes total thyroidectomy, radioiodine ablation, and the use of TSH-suppressing medications [34]. Studies indicate that for tumors ranging between 1 and 4 cm, the survival rates following TT and lobectomy are similar when various risk factors are taken into account [265]. In pediatric patients, lobectomy is often the preferred approach due to the lower likelihood of aggressive disease and the desire to preserve thyroid function [269]. The surgical strategy for FTC should be tailored to each individual, considering tumor characteristics, the patient’s age, and overall health to achieve the best outcomes and reduce the risk of complications [265]. Moreover, in the treatment of PTC with a dominant follicular variant (FVPTC), factors such as tumor size, extension outside the thyroid (extrathyroidal extension), and the patient’s gender are important. Research shows that for tumors smaller than 2 cm, lobectomy is typically sufficient, and more aggressive surgery is not necessary. However, for tumors larger than 2 cm, particularly those with extrathyroidal extension, TT is recommended, as it enhances survival rates [270].

#### 7.1.3. Medullary Thyroid Cancer

The treatment for MTC primarily involves surgery, with TT being the preferred option in most cases, even for smaller unilateral tumors. When MTC is confined to the thyroid, TT should be performed along with bilateral central LND at level VI. This approach is recommended because research has demonstrated that CLND increases the likelihood of a cure [271]. For patients with MTC that is confined to the neck but includes lateral neck lymph node metastases, total thyroidectomy, bilateral CLND, and selective lateral LND (covering at least levels II to V) are recommended. During this procedure, it is crucial to protect key anatomical structures such as the sternocleidomastoid muscle, internal jugular vein, and accessory nerve [272]. In individuals with RET mutations, particularly children and younger adults, prophylactic TT may be considered, depending on the type of mutation and the patient’s age. In adult patients with RET mutations, TT along with removal of the appropriate lymph nodes based on Ctn levels is generally advised [56]. Recently, for sporadic MTC that is clinically confined to a single thyroid lobe, a less invasive option such as hemithyroidectomy with or without diagnostic ipsilateral central LND has been suggested as a risk-reducing approach [273]. For hereditary MTC, the most effective strategy involves a DNA and biochemical-based approach, which limits prophylactic surgery to TT to minimize surgical risks before the tumor has spread beyond the thyroid capsule [273]. In some cases of sporadic MTC, thyroid lobectomy may be suitable, particularly if preoperative neck ultrasound shows no evidence of metastasis to the opposite thyroid lobe. Postoperative monitoring of Ctn levels is necessary, and if levels drop to undetectable levels after lobectomy, further surgery may not be required [274].

#### 7.1.4. Anaplastic Thyroid Cancer

**Figure 3:**
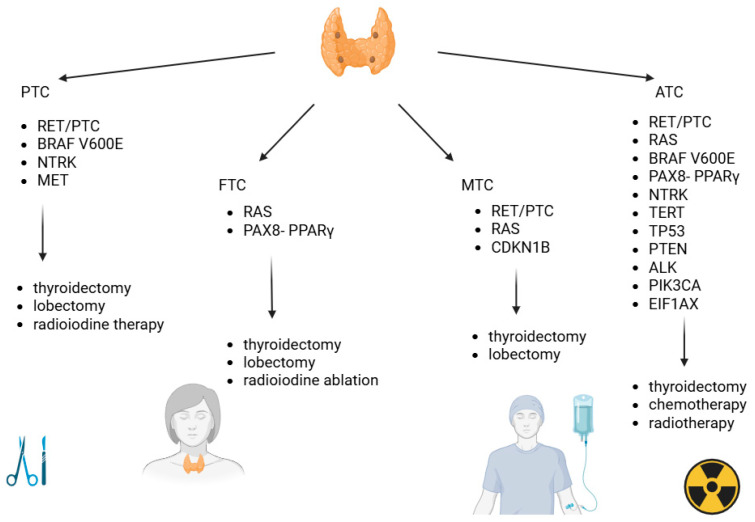
Thyroid cancers, genetics, and surgical treatment.

The treatment of choice for ATC is thyroidectomy, yet the majority of cases are diagnosed at an advanced stage, rendering them unresectable at presentation [275]. Surgery plays a crucial role in localized ATC, as surgical resection is the preferred treatment for this stage [71]. However, for patients with more advanced disease, especially stage IVC, TT is the preferred method because it has been shown to improve OS and DSS [276]. In some cases, debulking surgery—removal of part of the tumor—is considered. However, in stage IVC, this approach is generally contraindicated because it does not offer significant survival benefits and may result in severe complications, such as delaying the initiation of systemic treatments, including chemotherapy and radiotherapy [276]. The first step in treating ATC is often surgery for local control, particularly in patients with stage IVB. In such cases, surgery may prevent immediate death from asphyxiation; however, these patients often succumb to the disease due to lung metastases or other complications as their condition deteriorates [277]. Radiation therapy may also be implemented postoperatively, in addition to levothyroxine treatment, which is necessary for patients following thyroidectomy. This postoperative care is crucial to manage hormonal imbalance and other complications [275]. While radical surgery, involving the resection of organs responsible for speech and swallowing, is sometimes performed in certain cases, its benefits are questionable, even when clear margins are achieved. Most patients still experience poor outcomes despite undergoing surgery [278]. ATC is commonly diagnosed at an advanced stage, often with invasion into intrathoracic vessels or prevertebral tissues, which is frequently used as a criterion for unresectability. However, opinions on the precise definition of resectability vary, making treatment decisions more complex (Figure 3) [279].

### 7.2. Radioiodine Therapy

After undergoing thyroidectomy, patients diagnosed with PCT generally receive both radioactive iodine (RAI) therapy and lifelong thyroid hormone replacement. The primary role of RAI therapy is to eliminate any remaining thyroid tissue or cancer cells that may be left after surgery. It uses RAI isotopes to target and destroy these cells, enhancing the treatment’s post-surgery effectiveness [280]. Subsequently, thyroid hormone replacement is essential to compensate for the lost thyroid gland and maintain normal bodily functions. On the other hand, MTC, which arises from the thyroid’s parafollicular C cells, does not concentrate iodine, rendering RAI therapy ineffective. In these cases, alternative treatments must be considered, as RAI therapy and ablation fail to achieve the desired results. While RAI therapy provides significant benefits for many patients with DTC, it is not without its limitations. Resistance to RAI therapy develops in 33–50% of thyroid cancer patients over time, leading to poorer prognosis and reduced survival [281]. This resistance is often linked to factors such as the primary tumor exceeding 40 mm in size, extrathyroidal extension, age over 55 years, and early rises in TG levels, all of which strongly predict a suboptimal response to treatment [281]. For high-risk DTC patients, particularly those with lymph node involvement or distant metastases, RAI therapy has been associated with improved long-term survival, with patients suffering from PTC and metastases showing a clear benefit [282]. However, RAI therapy is ineffective in treating ATC due to the lack of iodine uptake and radioresistance in these tumors [283]. Certain conditions contraindicate the use of RAI therapy, including pregnancy, breastfeeding, the absence of iodine uptake in TC, and thyroid issues like nystagmus or eye diseases in Graves’ disease, as well as thyrotoxicosis [284]. For patients without metastases prior to the 123-I scan, an empirical radioiodine dose is typically administered [285]. The decision to proceed with RAI therapy is guided by the recurrence risk classification from the ATA, though it can be challenging to apply in clinical practice due to the multitude of variables involved. Additional diagnostic tests, such as measuring TG or TgAb and performing neck ultrasound, may help better identify suitable candidates for therapy [286]. To ensure the success of RAI therapy, a careful analysis of diagnostic data is essential in determining the appropriate dosage to minimize complications while maximizing treatment effectiveness. In some instances, patients with low or very low risk may avoid systemic RAI therapy and instead receive low-dose protocols, as evidenced by clinical trials like ESTIMABLE2 [286]. RAI therapy is most beneficial for high-risk patients, such as those with metastatic disease or aggressive tumors. However, its benefits remain less clear and are a subject of ongoing debate for intermediate-risk patients [287]. Efforts to restore sensitivity to RAI therapy by redifferentiating thyroid cancer cells have shown limited success. Although some treatments may increase iodine uptake, they do not always lead to a clinically meaningful response [288]. For those with RAI-refractory disease, which includes patients with structural disease despite adequate preparation for RAI therapy, redifferentiation strategies involving kinase inhibitors (e.g., BRAF, MEK, RET) can potentially improve iodine uptake and treatment outcomes [289]. Additionally, RAI therapy can lead to significant alterations in hematological parameters, such as a reduction in white blood cells, neutrophils, lymphocytes, platelets, and red blood cells. This change increases the risk of anemia, infection, and bleeding [290]. Furthermore, I131 (radioactive iodine) primarily induces thyroid cell death through apoptosis, although necrosis may also occur in some cases. This therapy also impacts the immune response, shifting it from autoantibody production to a chemokine-driven immune response that attracts immune cells to inflamed thyroid tissues [291]. In conclusion, RAI therapy remains a cornerstone in treating DTC, especially for high-risk patients. However, its effectiveness can be hindered by factors like resistance and the presence of complications. Thus, precise patient selection and individualized treatment plans are crucial for optimizing outcomes.

### 7.3. Chemotherapy

Chemotherapy is infrequently used in the treatment of TC, primarily in advanced cases such as ATC, which is more aggressive and does not respond well to conventional treatments [292]. In these situations, when surgery or RAI ablation does not yield satisfactory results, chemotherapy may become the preferred treatment option [292]. Although chemotherapy plays a limited role in TC treatment, it is mainly employed in ATC, where chemotherapy alone has shown limited success [293]. Common chemotherapy drugs used in ATC treatment include doxorubicin, cisplatin, and taxanes such as docetaxel and paclitaxel [293]. Despite their relatively widespread use, these medications do not substantially improve patient survival [294]. Moreover, chemotherapy combinations like doxorubicin and cisplatin often come with significant side effects, including hematological and gastrointestinal issues [295]. However, the combination of doxorubicin and cisplatin tends to produce a higher response rate than doxorubicin alone. In one study, 20% of patients showed a partial response, and 30% had stable disease. The median progression-free survival was 6 months, and the overall survival was 9 months, with tolerable side effects [296]. Doxorubicin chemotherapy remains one of the few palliative options for patients with advanced or metastatic thyroid cancer resistant to RAI treatment. Administering doxorubicin at a dose of 60 mg/m^2^ every three weeks can result in disease stabilization, which, given the aggressive nature of the tumor, can be considered a therapeutic success [297]. However, chemotherapy for TC is generally of limited effectiveness, especially in radioiodine-resistant cases [298].

### 7.4. Immunotherapy

**Figure 4:**
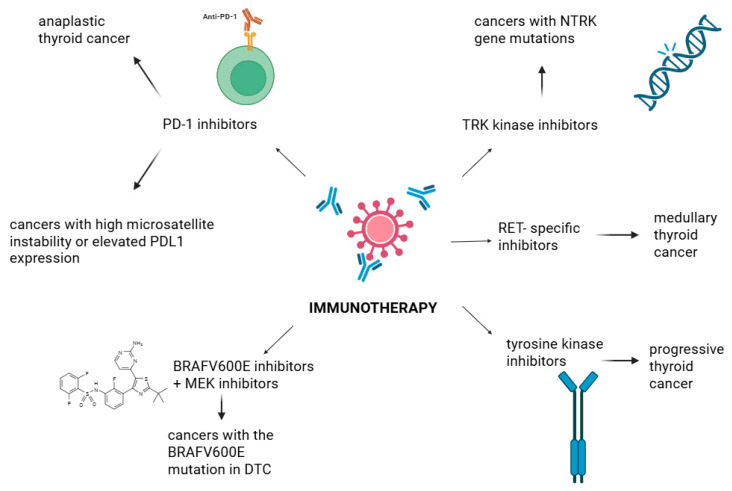
Immunotherapy in thyroid cancers.

Recent advancements in TC treatment focus on the use of immunotherapies and targeted therapies. Immunotherapies, such as PD-1 inhibitors (e.g., pembrolizumab and spartalizumab), enhance the patient’s immune system to target the tumor, especially in cases where the cancer is resistant to other treatments. These therapies are particularly effective for cancers with high microsatellite instability or elevated PDL1 expression, as well as in ATC [299]. Immunotherapy’s effectiveness varies by tumor type, with ATC showing a better response due to its higher mutational load, in contrast to PCT or FTC, which have a lower mutational load and PD-L1 expression [300]. Targeted therapies are essential for treating rare and aggressive TC forms. Tyrosine kinase inhibitors (TKIs) such as sorafenib, lenvatinib, vandetanib, and cabozantinib have been approved for treating progressive TC. Clinical trials have demonstrated their effectiveness in improving progression-free survival and treatment response rates (ORR) [301]. However, while TKIs are effective, they can cause specific side effects such as liver issues, gastrointestinal problems, hypertension, proteinuria, and fatigue, though they avoid typical chemotherapy side effects like hair loss and nausea [302]. In addition to TKIs, treatments for specific mutations, such as TRK kinase inhibitors (larotrectinib and entrectinib), have shown success in treating cancers with NTRK gene mutations [133]. BRAFV600E inhibitors, such as vemurafenib, and their combination with MEK inhibitors (dabrafenib + trametinib), are particularly effective for patients with the BRAFV600E mutation in DTC [303]. However, the combination of dabrafenib and trametinib, while highly effective, can lead to more side effects, particularly in older patients, causing some to stop treatment [303]. Genetic factors can limit the effectiveness of these therapies. For example, mutations such as V804M-RET in MTC can lead to resistance to certain drugs [304]. Although tyrosine kinase inhibitors can stabilize the disease, they often do not result in complete remission [304]. In cases of unresectable MTC, systemic therapies like RET-specific inhibitors (pralsetinib and selpercatinib) or TKIs such as cabozantinib and vandetanib should be used. For tumors with microsatellite instability-high status or mismatch repair deficiencies, pembrolizumab therapy is also an option [304]. Targeted therapies continue to evolve, with new drugs like pralsetinib and selpercatinib focusing on RET gene mutations and entrectinib, larotrectinib, and repotrectinib targeting NTRK gene mutations. The use of BRAF inhibitors like vemurafenib and dabrafenib, MEK inhibitors such as trametinib, and anti-angiogenic multi-targeted kinase inhibitors (e.g., lenvatinib and sorafenib) is also becoming more widespread [305]. Ongoing monitoring is crucial in these treatments. Follow-up methods for DTC include serum TG measurements, neck ultrasonography, and occasionally I-131 whole-body scintigraphy [306]. Additionally, US-guided FNA-Tg has been shown to be a valuable tool for monitoring DTC patients, regardless of TSH levels or the presence of TgAb [307]. In summary, a modern approach to TC treatment involving immunotherapy and targeted therapies offers significant promise, especially for advanced or resistant cancer cases. However, these treatments’ success depends on genetic mutations, and close monitoring is essential to manage side effects and assess their ongoing effectiveness (Figure 4).

### 7.5. Comparison of First-Line and Second-Line Therapies

First-line therapies for TC typically include surgical resection, radioiodine ablation (RAI) (mainly in differentiated thyroid cancer (DTC), and TSH suppression [107,121]. These methods, especially when used in early and well-differentiated tumors such as PTC and FTC, are often curative therapies [107,110,115,121]. Total thyroidectomy with bilateral lymph node dissection remains the recommended approach in children with DTC, while thyroid lobectomy is commonly used in minimally invasive FTC [110,121]. In the case of MTC, surgical treatment—particularly TT with central neck dissection—is the mainstay of therapy, especially when the disease is limited to the thyroid gland [127]. Similarly, ATC, which is highly aggressive, is initially treated with thyroidectomy if possible, often followed by postoperative radiotherapy and levothyroxine therapy [107]. However, there are cases where the disease becomes refractory to first-line interventions—such as RAI-refractory DTC, unresectable tumors, or advanced/metastatic disease—in which case second-line therapies are required. RAI resistance develops in approximately 33–50% of patients with DTC over time, and this reduces survival and treatment efficacy [139]. In such cases, systemic therapies become necessary. These include tyrosine kinase inhibitors (TKIs) such as sorafenib and lenvatinib for progressive, RAI-refractory DTC and vandetanib or cabozantinib for advanced MTC [171]. In addition, newer RET-specific inhibitors such as pralsetinib and selpercatinib have shown efficacy in unresectable or metastatic MTC with RET mutations [171]. In ATC, BRAF and MEK inhibitors (e.g., dabrafenib and trametinib) are considered second-line options, particularly in BRAF-mutated tumors, with objective response rates above 60% [171]. Second-line treatments are often associated with a higher toxicity burden and a lower likelihood of achieving complete remission compared with first-line treatment. For example, lenvatinib in RAI-refractory DTC results in a median progression-free survival of approximately 18 months, which contrasts with the potential for complete remission with initial RAI therapy in early stage disease [139]. Furthermore, efforts to redifferentiate thyroid cancer cells to restore iodine sensitivity have had limited clinical impact, despite theoretical promise [146,147]. Chemotherapy, especially with doxorubicin, remains a palliative option in advanced, refractory cases [146]. As systemic treatment options evolve, early integration of targeted or immune-based therapies is increasingly being considered for aggressive variants such as tall cell thyroid cancer or poorly differentiated thyroid cancer (Table 5) [171].

### 7.6. Molecularly Targeted Therapies in Thyroid Cancer: Recent Advances

Recent advances in targeted therapies have resulted in the approval of new molecularly targeted drugs such as selpercatinib and pralsetinib, which have shown high efficiency in patients with MTC with RET mutations and DTC with RET fusions [308]. Both medications are selective RET kinase inhibitors that have been approved following the findings of trials such as LIBERTTO-001 (NTC03157128) and ARROW (NTC03037385) [309]. Concurrently, RET inhibitors such as pralsetinib have demonstrated success in patients with RET-altered thyroid tumors, including those who have received prior treatment [310]. Furthermore, PD-1 inhibitors like spartalizumab, when taken with lenvatinib, have demonstrated encouraging outcomes in the treatment of anaplastic thyroid carcinoma, a particularly aggressive kind of thyroid cancer (Table 6) [311].

### 7.7. Considerations in Resource-Limited Settings

In resource-limited settings, the management of thyroid cancer poses significant challenges due to various limitations in diagnostic tools, treatment modalities, and surgical expertise [313]. A lack of access to advanced diagnostic technologies such as molecular testing (e.g., BRAF or RET mutation panels), CT, and MRI is another intractable problem [314]. As a result, physicians often rely on more basic imaging modalities such as ultrasound, which are more affordable and available in many centers. However, they may not provide the same level of detail as more advanced methods. Additionally, essential therapies such as I-131 therapy, which is key in the treatment of DTC [315], may be unavailable or prohibitively expensive. Both targeted therapies and genetic testing are essential for personalized treatment plans but are often out of reach, leading to diagnostic limitations [316]. Surgical care may also be compromised, as a shortage of skilled endocrine surgeons increases the risk of suboptimal surgery, potentially affecting prognosis [317]. Furthermore, inadequate access to postoperative monitoring tools such as serum TG testing or high-quality ultrasound impedes the detection of recurrences and long-term management [161]. As a result, treatment protocols may be simplified, and lobectomy may be preferred over total thyroidectomy to reduce the dependence on lifelong hormonal therapy, which may not be consistently available [318]. In such circumstances, telemedicine consultations, regional centers of excellence, and international outreach programs play a key role in improving outcomes by facilitating access to both medical expertise and essential medications.

## 8. Future Perspectives

Future perspectives in TC therapy focus on the integration of genomic profiling and liquid biopsy to guide personalized treatment strategies and overcome resistance to some existing therapies. Continued research into novel actionable mutations and immune targets is essential to expand therapeutic options and improve outcomes for patients with advanced disease [319]. In the near future, artificial intelligence (AI) technology may be helpful in accessing the morphological and molecular features of TC. Recent advancements in AI have significantly enhanced the diagnostic accuracy of thyroid nodules by leveraging quantitative morphological and cytological features, aiding in the differentiation of challenging entities such as follicular adenoma, follicular carcinoma, and variants of papillary thyroid carcinoma. Furthermore, the integration of AI with molecular testing, including gene panels and protein-based classifiers, holds promise for improving risk stratification, personalized treatment planning, and the early identification of aggressive disease, thus setting the stage for more precise and individualized TC management [320]. Moreover, further research on TC risk factors is vital to elucidate the impact of endocrine-disrupting chemicals (EDCs) on the incidence and progression of TC and thyroid-related disorders. Scientists should focus on the role of various classes of EDCs, including heavy metals, cosmetic ingredients, industrial chemicals, pesticides, herbicides, pharmaceuticals, and both synthetic and naturally occurring hormones. Comprehensive studies are needed to better understand their mechanisms of action, exposure pathways, and potential effects on thyroid function and carcinogenesis [321]. Future perspectives of TC management also highlight the expanding role of PET/CT, particularly in identifying recurrent or metastatic disease in patients with non-iodine-avid tumors or elevated thyroglobulin levels. Advancements in novel radiotracers and theragnostic applications may further enhance its clinical utility, supporting more personalized and effective treatment strategies, though efforts to improve accessibility, cost-efficiency, and standardization remain essential [322].

## 9. Conclusions

Thyroid cancer has become a growing global health issue, with its incidence increasing notably in recent years. This rise is largely attributed to increased exposure to environmental factors such as chemicals, radiation, and pollution, especially in areas with a higher HDI. Despite the growing number of diagnoses, death rates have remained relatively stable, suggesting that while thyroid cancer is generally treatable, challenges persist in its early detection and management. The disease’s complex epidemiology shows a higher prevalence among women and younger populations, underscoring the need for targeted treatment approaches for different cancer subtypes, such as papillary, follicular, medullary, and anaplastic thyroid cancers. Recognizing key risk factors such as physical inactivity, obesity, radiation exposure, and genetic mutations is essential for understanding thyroid cancer’s development. Specifically, mutations in genes like BRAF, RAS, RET, and NTRK are crucial in assessing disease severity and prognosis. Genetic testing, especially for BRAF V600E and RET mutations, plays a critical role in tailoring treatment plans, offering better predictions of disease behavior and aiding therapeutic decisions. Various biomarkers, including TG, Ctn, CEA, and Pct, are vital for diagnosing and monitoring thyroid cancer. These markers are useful for early detection, tracking disease progression, and evaluating treatment effectiveness, particularly in differentiated and medullary thyroid cancers. The emerging role of microRNAs as exosomal biomarkers shows promising potential for enhancing diagnostic accuracy and patient classification. Although traditional treatments such as surgery and RAI therapy remain foundational, their effectiveness depends on cancer subtype, tumor size, and genetic characteristics. RAI therapy works well for many differentiated thyroid cancers but faces resistance, particularly in medullary and anaplastic types, making alternative treatments like chemotherapy and targeted therapies increasingly important. Immunotherapies and TKIs have shown potential in cases resistant to standard treatments, though their success relies heavily on genetic factors and precise patient selection. In conclusion, continued progress in genetic profiling, biomarker discovery, and personalized treatment strategies is crucial to improving thyroid cancer outcomes. While early detection and conventional therapies benefit many patients, those with advanced or resistant forms require more innovative treatments targeting specific genetic mutations for better management. The future of thyroid cancer care will likely combine genetic insights, novel therapies, and individualized approaches to optimize patient outcomes and mitigate the impact of this growing disease.

## Figures and Tables

**Table 1:** The most common mutations in chosen types of thyroid cancer.

Type of Cancer/Mutation	Papillary (PTC)	Medullary (MTC)	Follicular (FTC)	Anaplastic (ATC)
RET/PTC	+	+	−	+
RAS	−	+	+	+
BRAF V600E	+	−	−	+
PAX8-PPARγ	−	−	+	+
NTRK	+	−	−	+
MET	+	−	−	−
CDKN1B	−	+	−	
TERT	−	−	−	+
TP53	−	−	−	+
PTEN	−	−	−	+
ALK	−	−	−	+
PIK3CA	−	−	−	+
EIF1AX	−	−	−	+

**Table 2:** Comparison of four major types of thyroid cancer.

Cancer Type	PTC	FTC	MTC	ATC
Incidence (% of all TC cases)	90%	20%	1–5%	2%
Age	30–50 years old	40–50 years old	40–60 years old	Over 65 years old
Prognosis	Relatively mild cancer	80% mild; 20% aggressive	Poor prognosis	Very poor prognosis
Metastatic potential	70% of patients present metastases to the lymph nodes of the neck	Metastases present more often than in PTC cases	High probability of metastasis	20–50% of ATC cases present metastases

**Table 3:** Diagnostic techniques for thyroid cancer. Overview of diagnostic techniques used in the evaluation of thyroid cancer, summarizing their diagnostic value, limitations, and current recommendations based on the latest guidelines from the American Thyroid Association (ATA) and the National Comprehensive Cancer Network (NCCN). The table aims to provide a systematic comparison of conventional and advanced methods, with particular emphasis on their role in assessing malignancy risk and guiding clinical decision-making in indeterminate and advanced cases.

Diagnostic Technique	Diagnostic Value	Limitations	Recommendations (ATA, NCCN)
Ultrasound (US)	Primary method for evaluating thyroid nodules, detects features suspicious for malignancy (e.g., microcalcifications) [131]	May not be sufficient for definitive diagnosis, especially in indeterminate nodules [136]	Recommended as the first step in diagnosis (ATA, NCCN) [133]
Fine-needle aspiration biopsy (FNAB)	Cytological evaluation helps assess malignancy risk, particularly in indeterminate nodules [133]	Results can be indeterminate for Bethesda III/IV nodules [137]	Essential for indeterminate nodules (ATA, NCCN) [135]
Molecular genetic testing	Increases diagnostic accuracy, particularly for Bethesda III/IV nodules, aiding in treatment planning [137]	High cost, availability may be limited, not always accessible in every center [137]	Recommended for indeterminate nodules (ATA, NCCN) [137]
Single-photon emission computed tomography (SPECT)	Helps assess malignant changes and potential metastasis [135]	Requires specialized equipment, less useful for evaluating small nodules [136]	Recommended in advanced disease, particularly for metastasis assessment (ATA) [71]
Computed tomography (CT)	Useful for assessing metastasis, especially in advanced cases [71]	Limited value for diagnosing early thyroid tumors [135]	Recommended for metastasis assessment (NCCN) [71]
Magnetic resonance imaging (MRI)	Helps differentiate between benign and malignant nodules, especially in advanced disease [134]	Expensive, time-consuming, requires specialized equipment [134]	Recommended for assessing local invasion and metastasis (NCCN) [134]
PET/CT, PET/MRI	Advanced techniques useful for assessing poorly differentiated or dedifferentiated TC [142,143]	Expensive, limited availability in some centers [142,143]	Recommended in advanced TC, particularly for staging and follow-up (ATA, NCCN) [142,143]
High-throughput sequencing (NGS, WES, RNA-Seq)	Allows for the complete profiling of genetic changes (mutations, fusions, expression), which enhances diagnosis and tailored therapy selection in intermediate or aggressive situations [151,152,154]	Expensive, requires bioinformatics support, limited access in some contexts [156]	Suggested in cases of ambiguous cytology or probable advanced illness (ATA, NCCN) [153,155]

**Table 4:** Diagnostic and prognostic markers in thyroid cancer subtypes and their clinical application. A summary of significant diagnostic and prognostic biomarkers in thyroid cancer, organized by histological subtype and clinical applicability. The table summarizes the primary value of each marker—such as diagnosis, monitoring, or risk assessment—as well as any pertinent limitations, such as specificity, variability, or clinical application. The purpose of this overview is to clarify the role of each biomarker in subtype-specific thyroid cancer therapy.

Marker	Subtype(s)	Clinical Application	Limitations/Notes
Thyroglobulin (Tg)	PTC, FTC (DTC) [157,158,159,160]	Recurrence detection, residual disease monitoring, preoperative risk assessment [158,161,165]	Affected by TgAb; also secreted by benign thyroid remnants [163,164]
Calcitonin (Ctn)	MTC [167,168]	Early diagnosis, postoperative monitoring, biochemical cure assessment [169,171,172,173,175]	Elevated in non-malignant conditions; rapidly degraded in plasma [168,170]
Carcinoembryonic antigen (CEA)	MTC [177,179,181]	Disease progression monitoring, aggressiveness estimation [179,183,186,187,189]	Non-specific; influenced by liver function; high levels correlate with poor prognosis [180,181,185,186]
Procalcitonin (Pct)	MTC [190,198]	Tumor size correlation, progression monitoring; adjunct to Ctn and CEA [190,198,199]	Elevated in infections, trauma, surgery; not exclusive to C cells [191,192,193,195]
TSH	PTC, FTC (DTC) [202,204]	Postoperative management, recurrence risk stratification [205,206,208,209]	Oversuppression risks (e.g., osteoporosis); complex relationship with recurrence [207,210,211]
miRNAs	PTC, FTC, ATC [212,217]	Diagnostic and prognostic value, patient stratification [216,220,221,224,225]	Experimental; conflicting evidence; requires clinical validation [218,219,222,223]
BRAF V600E	PTC, ATC [218,229,232]	Risk of lymph node metastasis, iodine-refractory tumor indication [227,228,232]	Not reliable as a sole prognostic marker in low-risk disease [230,231,233]
RAS mutations	FTC, PTC (follicular variant), ATC [234,236,237]	Diagnostic aid, metastatic risk, mortality prediction [239,240,241]	Common in follicular-pattern tumors; also present in benign lesions [238,242]
RET/PTC rearrangements	PTC (esp. radiation-induced), pediatric MTC [244,246,250]	Diagnostic relevance, aggressive behavior predictor, pediatric cases [249,250,251]	Present in both benign and malignant lesions; strongly linked to radiation exposure [252,253]

**Table 5:** Comparison of first-line and second-line therapies in thyroid cancer.

Type of Thyroid Cancer	First-Line Therapy	Second-Line Therapy
PCT	- Surgery: lobectomy or total thyroidectomy [107] - Postoperative radioactive iodine (RAI) therapy [138] - Thyroid hormone replacement [138]	- Redifferentiation (BRAF, MEK, RET inhibitors) [147] - Tyrosine kinase inhibitors (TKIs), immunotherapy in resistant cases [163,165]
FTC	- Surgery: lobectomy, isthmectomy, total thyroidectomy (TT) [117,121] - RAI therapy [121] - TSH suppression [121]	- Targeted therapies in resistant cases (TKI) [165]
MTC	- Surgery: total thyroidectomy (TT) + central lymph node dissection (CLND) [125,126,127]	- RET inhibitors (pralsetinib, selpercatinib) [164] - Tyrosine kinase inhibitors (TKIs): cabozantinib, vandetanib [165,170]
ATC	- Surgery (if operable) [131,132] - Radiotherapy [131] - Thyroid hormone replacement [131]	- Chemotherapy: doxorubicin, cisplatin, paclitaxel [150,151,160] - Immunotherapy: PD-1 inhibitors (pembrolizumab, spartalizumab) [163]
RAI-refractory DTC	- Radioactive iodine (RAI) therapy (if still effective) [138,140]	- Kinase inhibitors: sorafenib, lenvatinib [165] - Redifferentiation: BRAF/MEK/RET inhibitors [146,147]
RET/NTRK mutated cancers	- Surgery (if resectable)	- RET inhibitors: pralsetinib, selpercatinib [170,171] - NTRK inhibitors: larotrectinib, entrectinib, repotrectinib [167,171]

**Table 6:** Selected current clinical trials [312].

Study ID (NCT)	Drug Combination	Molecular Target	Thyroid Cancer Type	Phase	Status
NCT04760288	Pralsetinib vs. standard care	RET	RET-mutant MTC	3	Recruiting
NCT04006676	Pralsetinib	RET	MTC	1/2	Active, not recruiting
NCT03954791	Entrectinib	NTRK	NTRK fusion-positive thyroid cancers	2	Completed
NCT04222972	Spartalizumab + lenvatinib	PD-1 + VEGF	ATC	1/2	Recruiting
NTC03157128	Selpercatinib	RET inhibitor	MTC, DTC	1/2	Completed
NTC03037385	Pralsetinib	RET inhibitor	MTC, DTC	1/2	Completed

## Data Availability

Not applicable.

## Abbreviations

The following abbreviations are used in this manuscript:

131I	iodine-131
AHR	aryl hydrocarbon receptor
AI	artificial intelligence
AP1	activator protein 1
ATA	American Thyroid Association
ATC	anaplastic thyroid cancer
BMI	body mass index
CEA	carcinoembryonic antigen
CGRP	calcitonin gene-related peptide
CRP	C-reactive protein
CT	computed tomography
Ctn	calcitonin
DC	dendritic cell
DSS	disease-specific survival
DSV	diffuse sclerosing variant
DTC	differentiated thyroid cancer
EDCs	endocrine-disrupting chemicals
ERα	estrogen receptor alpha
ERβ	estrogen receptor beta
FMTC	familial medullary thyroid cancer
FNA	fine-needle aspiration
FNAB	fine-needle aspiration biopsy
FNAC	fine-needle aspiration cytology
FT3	free triiodothyronine
FT4	free thyroxine
FTC	follicular thyroid carcinoma
FTC	follicular thyroid cancer
FVPTC	follicular variant of papillary thyroid carcinoma
FVPTC	PTC with a dominant follicular variant
HDI	Human Development Index
HT	Hashimoto thyroiditis
HV	hobnail
IGF-1	insulin-like growth factor 1
LND	lymph node dissection
LNM	lymph node metastasis
MAPK	MAP kinase
MEN2	multiple endocrine neoplasia type 2
MEN4	multiple endocrine neoplasia type 4
MRI	magnetic resonance
MTC	medullary thyroid cancer
NF-kB	nuclear factor kappa-light-chain-enhancer of activated B
NIFTP	non-invasive follicular thyroid neoplasm with papillary-like nuclear features
NIS	sodium–iodide symporter
ORR	treatment response rate
OS	overall survival
PCR	reverse-transcriptase polymerase reaction
Pct	procalcitonin
PTC	papillary thyroid cancer
RAI	radioactive iodine
RF	radiofrequency
SHS	secondhand smoking
SPECT	single-proton emission computed tomography
SV	solid variant
T3	triiodothyronine
T4	thyroxine
TAMs	tumor-associated macrophages
TBG	thyroxine-binding globulin
TBSRTC	The Bethesda System for Reporting Thyroid Cytopathology
TC	thyroid cancer
TCV	tall cell variant
TG	thyroglobulin
TKIs	tyrosine kinase inhibitors
TME	tumor microenvironment
TNF	tumor necrosis factor
TSH	thyroid-stimulating hormone
TSHRs	TSH receptors
TT	total thyroidectomy
TgAb	anti-TG antibody
US	ultrasonography
VIP	vasoactive intestinal peptide
lncRNAs	long non-coding RNAs
mRNA	messenger RNA
miRNA	microRNA
